# Breast Metastasis From Cervical Small Cell Neuroendocrine Carcinoma Mimicking Primary Breast Cancer

**DOI:** 10.7759/cureus.115684

**Published:** 2026-09-02

**Authors:** Gladys Bruyninx, Erica Patocskai, Lilia Maria Sanchez, Mona El Khoury, Ahmad Kaviani

**Affiliations:** 1 General Surgery, Fondation du CHUM, Montreal, CAN; 2 Surgical Oncology, Université de Montréal, Montreal, CAN; 3 Pathology, Centre Hospitalier de l’Université de Montréal, Montreal, CAN; 4 Radiology, Centre Hospitalier de l’Université de Montréal, Montreal, CAN; 5 Surgical Oncology, Centre Hospitalier de l'Université de Montréal, Montreal, CAN

**Keywords:** breast metastasis, cervical carcinoma, immunohistochemistry (ihc), mastectomy (d008408), neuroendocrine carcinoma (nec)

## Abstract

We describe a case of breast metastasis originating from a small-cell neuroendocrine carcinoma (SCNEC) of the uterine cervix, an uncommon presentation that can be difficult to distinguish from a primary breast malignancy. We report a 29-year-old woman treated three years earlier for FIGO stage IB2 cervical SCNEC with chemotherapy, surgery, and radiotherapy, who presented with a 1.5-cm mass in the left breast. Imaging suggested a primary breast malignancy, but her oncologic history raised concern for metastasis. Core-needle biopsy showed a high-grade small-cell neoplasm expressing synaptophysin, chromogranin A, and CD56, with a high Ki-67 index (>80%); breast-lineage markers, including GATA3 and mammaglobin, were negative. Whole-body fluorodeoxyglucose positron emission tomography-computed tomography (FDG PET-CT) showed an isolated hypermetabolic breast lesion without other disease. Nipple-sparing mastectomy with immediate reconstruction was performed, followed by platinum-etoposide chemotherapy; at 18 months, there was no evidence of recurrence. Breast metastasis from cervical SCNEC can mimic primary breast cancer; diagnosis relies on integrating clinical history, imaging, and immunohistochemistry. Selected patients with isolated disease may benefit from individualised multimodal therapy.

## Introduction

Neuroendocrine carcinomas of the uterine cervix are rare, aggressive tumours, comprising approximately 0.9-1.5% of cervical cancers, characterised by early lymphatic and haematogenous dissemination [1,2]. Despite radical surgery, chemotherapy, and radiotherapy, the prognosis remains poor, with high recurrence even in early-stage disease [2].

Breast metastases from extramammary malignancies are also rare, comprising fewer than 2% of breast tumours. The most frequent primary sites are melanoma, lung, and gastrointestinal cancers, whereas gynaecologic primaries are uncommon, and breast metastasis from cervical small-cell neuroendocrine carcinoma (SCNEC) has only rarely been reported [3-6]. Because such lesions may resemble primary breast carcinoma clinically and radiologically, distinguishing them is essential. Diagnosis depends on correlating the oncologic history with morphology and immunohistochemistry [7]. We report an isolated breast metastasis from cervical SCNEC occurring three years after definitive treatment of the primary tumour.

## Case presentation

A 29-year-old woman (G5 P3 L3 A1) was diagnosed in 2020 with a high-grade SCNEC of the uterine cervix at 26 weeks' gestation, after presenting with a friable, exophytic cervical mass. Biopsy showed small-cell morphology with immunopositivity for synaptophysin, chromogranin A, and CD56. Pelvic MRI demonstrated a 16 x 22 mm cervical lesion without parametrial infiltration or lymphadenopathy, consistent with FIGO 2018 stage IB2. Given the gestational age and tumour aggressiveness, she received two cycles of neoadjuvant carboplatin-etoposide during pregnancy. Caesarean delivery was scheduled four weeks after the final chemotherapy cycle to reduce the risk of foetal thrombocytopenia. At 35 weeks' gestation, following two doses of antenatal betamethasone for foetal lung maturity, she underwent a scheduled classic caesarean section, followed in the same operative setting by radical hysterectomy, bilateral salpingo-oophorectomy, and bilateral pelvic lymphadenectomy. Final surgical pathology confirmed high-grade SCNEC with a stable (mismatch repair-intact) profile, a depth of invasion of 6.8 mm (60% of cervical stromal thickness), a tumour measuring 4 cm, extensive lymphovascular space invasion, and perineural invasion; surgical margins and lymph nodes were negative, and the tumour was reclassified as FIGO 2018 stage IB3. Based on Sedlis criteria, she received adjuvant pelvic radiotherapy (45 Gy in 25 fractions via intensity-modulated radiation therapy (IMRT), with a boost to the vaginal vault to 50.4 Gy) together with two cycles of adjuvant cisplatin-etoposide chemotherapy. High-risk human papillomavirus (HPV) testing of the cervical tumour was positive, consistent with its role in the pathogenesis of the malignancy. There was no relevant family history of breast or gynaecologic malignancy.

After about three years in remission, she presented in 2023 with a firm, painless, mobile 1.5-cm mass in the upper inner quadrant of the left breast, without skin or nipple changes or palpable axillary nodes. Mammography showed an oval, high-density mass with indistinct margins and no microcalcifications (Figure 1), and ultrasonography showed a solid, hypoechoic mass with indistinct margins and posterior acoustic enhancement, assigned BI-RADS category 4C (Figure 1).

**Figure 1:**
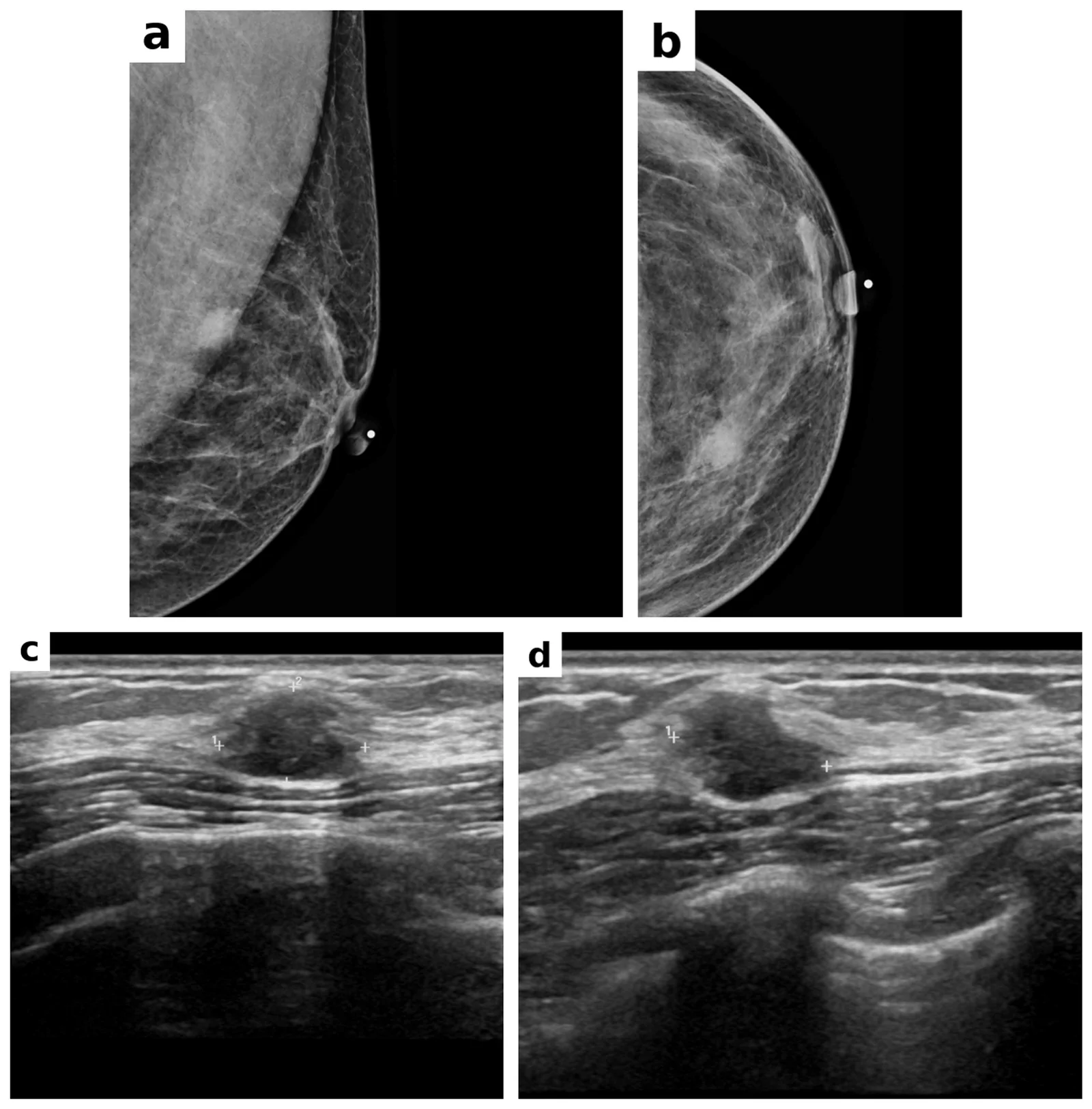
Breast imaging. (a) Left mediolateral-oblique and (b) craniocaudal mammograms show an oval, high-density mass with indistinct margins in the upper inner quadrant of the left breast (approximately 1.3 x 0.8 cm), without architectural distortion or suspicious microcalcifications. (c) Radial and (d) antiradial ultrasound images show a solid, hypoechoic mass with indistinct margins and posterior acoustic enhancement.

Although imaging suggested a primary breast malignancy, her history of cervical SCNEC raised concern for metastasis, and core-needle biopsy revealed a high-grade small-cell neoplasm. Staging whole-body fluorodeoxyglucose positron emission tomography-computed tomography (FDG PET-CT) demonstrated an isolated hypermetabolic left-breast lesion (SUVmax 5.0) with no other disease, including no axillary or pulmonary lesions (Figure 2).

**Figure 2:**
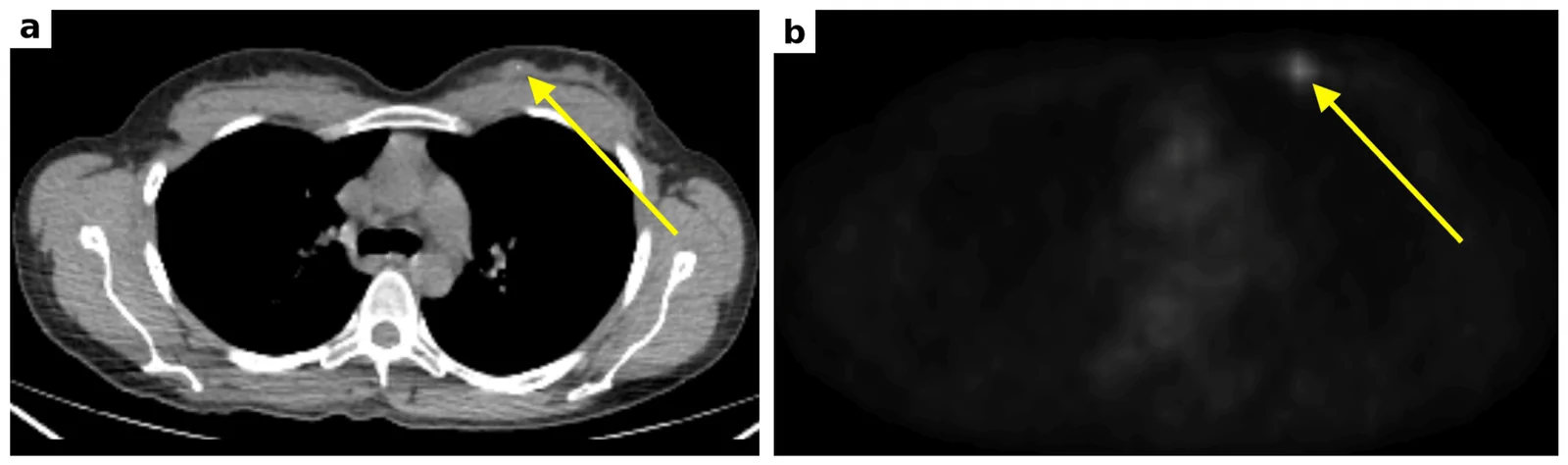
Fluorodeoxyglucose positron emission tomography-computed tomography (FDG PET-CT). (a) Axial CT and (b) axial fused PET-CT images at the same level show an intensely hypermetabolic focus in the upper inner quadrant of the left breast (arrows), corresponding to the biopsy-proven metastatic small-cell neuroendocrine carcinoma; the biopsy marker clip is visible within the lesion. No other hypermetabolic disease was identified in the remainder of the whole-body study. PET-CT: Positron emission tomography-computed tomography.

Histologically, the tumour formed infiltrative solid sheets and nests of cells with scant cytoplasm, finely granular chromatin, a high nuclear-to-cytoplasmic ratio, nuclear moulding, and frequent mitoses (Figure 3). Immunohistochemistry showed strong synaptophysin, chromogranin A, and CD56 positivity; a high Ki-67 index (>80%); strong, diffuse p16; and nuclear TTF-1 (Figure 3). Hormone receptors (oestrogen receptor, progesterone receptor) and HER2 were negative, as were the breast-lineage-specific markers GATA3 and mammaglobin; all markers were reported together as a panel on the pathology report. Direct comparison with the archived 2020 cervical specimen confirmed concordant morphology and immunophenotype, supporting histological identity between the two tumours. These features were consistent with metastatic SCNEC rather than a primary breast tumour.

**Figure 3:**
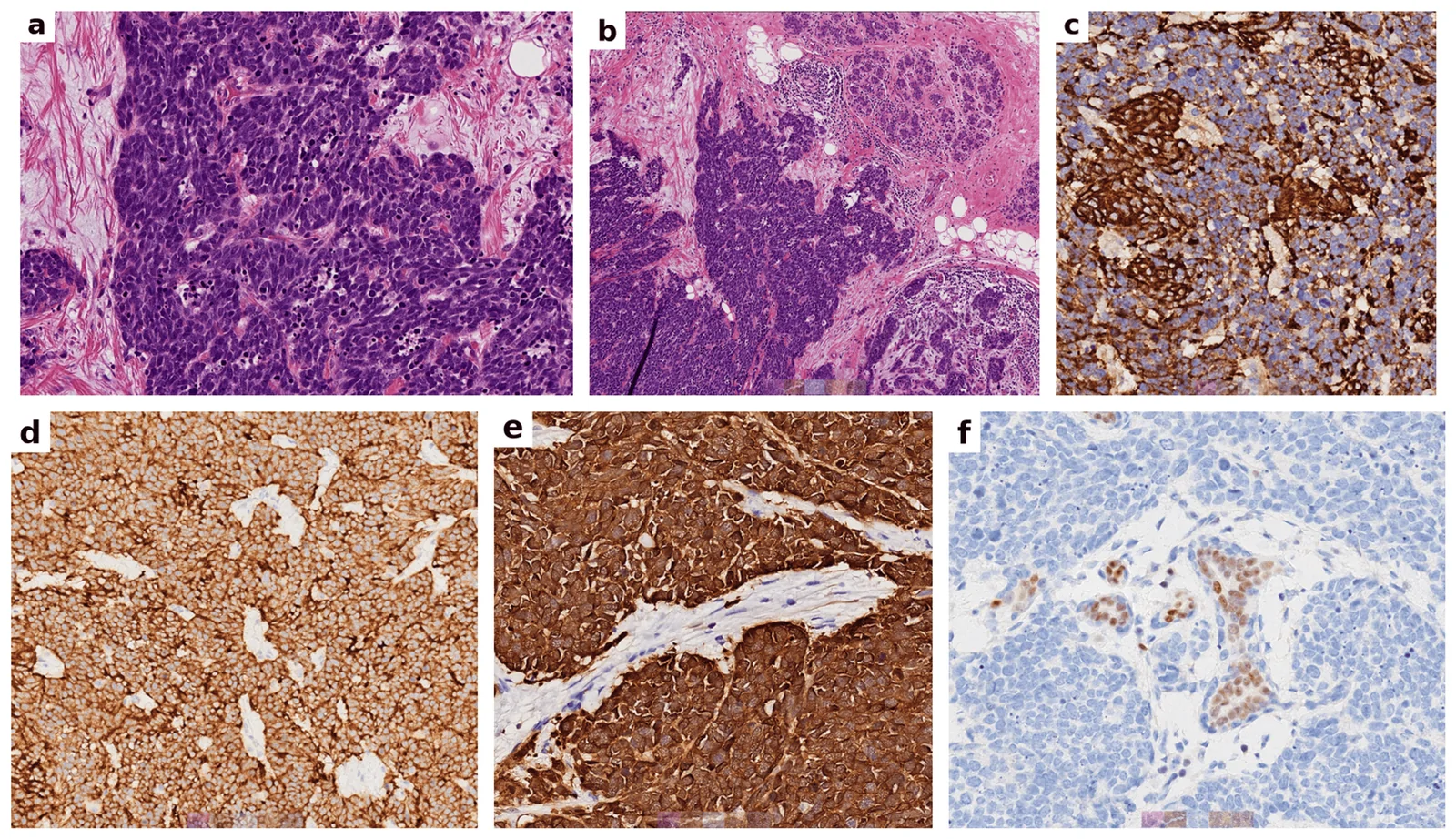
Histopathology and immunohistochemistry of the metastatic small-cell neuroendocrine carcinoma (SCNEC). (a) A low-power (haematoxylin and eosin (H&E), x100) view shows infiltrative tumour arranged in solid sheets and nests. (b) A high-power (H&E, x200) view shows small cells with scant cytoplasm, finely granular chromatin, high nuclear-to-cytoplasmic ratio, nuclear moulding, and frequent mitoses. (c) Chromogranin A immunohistochemistry (x200) shows diffuse positivity. (d) p16 immunohistochemistry (x200) shows strong, diffuse nuclear and cytoplasmic positivity. (e) TTF-1 immunohistochemistry (x200) shows nuclear positivity, considering the prior cervical primary. (f) Oestrogen receptor immunohistochemistry (x400) shows no tumour cell expression.

At multidisciplinary review, the large tumour-to-breast ratio made breast-conserving surgery likely to yield poor cosmesis. She underwent a left nipple-sparing mastectomy with immediate reconstruction using a tissue expander on the affected side and a contralateral implant for symmetry. Axillary surgery was omitted given the absence of axillary avidity on PET-CT and the metastatic, rather than primary, nature of the disease. Final pathology confirmed metastatic SCNEC measuring 1.8 cm, with negative margins. She then received four cycles of platinum-etoposide chemotherapy; cisplatin was switched to carboplatin after the second cycle because of ototoxicity. Treatment was otherwise well tolerated, with no other major adverse events reported. Surveillance consisted of serial CT of the chest, abdomen, and pelvis every six months, with an initial brain CT at the first follow-up visit. At 18 months after surgery, she remained free of clinical or radiologic recurrence (Figure 4).

## Discussion

SCNEC of the cervix is highly aggressive, with early dissemination and frequent recurrence despite multimodal treatment [2]. Most cases, including our patient's tumour, are associated with high-risk HPV, particularly HPV-18. Biologically, these tumours resemble pulmonary small-cell carcinoma and share its poor outcomes [7-9].

Metastasis to the breast from extramammary malignancies is likewise uncommon, accounting for fewer than 2% of breast malignancies. The most frequent primary sites include melanoma, lung, and gastrointestinal cancers, whereas gynaecologic primaries are rare. Metastatic cervical SCNEC to the breast is particularly uncommon and has been reported only in isolated cases [3,5,6]. Such metastases may mimic a primary breast carcinoma clinically and radiologically, typically presenting as a solitary, circumscribed mass [4]. Absence of an in situ component and negativity for breast-lineage markers (GATA3, mammaglobin, GCDFP-15), together with expression of neuroendocrine markers (synaptophysin, chromogranin A, CD56), support a metastatic neuroendocrine origin [7,8]. A key differential diagnosis is primary small-cell carcinoma of the breast, which is exceptionally rare [10]. In our patient, however, the history of cervical SCNEC, concordant morphology and immunophenotype, absence of another primary lesion on FDG PET-CT, and lack of breast-lineage markers collectively favoured metastatic disease. TTF-1 positivity, although often linked to pulmonary origin, can occur in extrapulmonary SCNEC; combined with strong p16 expression, the neuroendocrine phenotype, and no pulmonary disease, it remained consistent with a cervical source.

This case is distinctive for its long disease-free interval and isolated, oligometastatic presentation. In previous reports, breast involvement has signalled aggressive systemic relapse. Kwak described a breast metastasis three months after treatment of the primary, followed by rapid progression despite surgery and chemotherapy [5], and Wannasri et al. reported breast involvement one year after chemoradiation alongside lung, skin, peritoneal, and nodal disease, with a rapidly fatal course [11]. By contrast, our patient had no extramammary disease on PET-CT and achieved durable remission at 18 months after treatment [12]. While systemic therapy remains the standard for disseminated SCNEC, selected patients with limited metastatic burden may benefit from individualised management combining local and systemic treatment; surgery was chosen here given the isolated lesion. Axillary staging was omitted based on the absence of axillary avidity on PET-CT and the metastatic nature of the disease; this is consistent with a review of 38 historical cases of cervical cancer metastatic to the breast, in which most patients were managed with systemic chemotherapy, only two underwent any form of breast surgery, and axillary staging was not reported in any case [13].

Previously reported cases of cervical small-cell carcinoma with breast metastasis, compared with the present case, include:

Viswanathan et al. [14] (2004): A 21-patient outcome series of cervical SCNEC examining patterns of recurrence; one patient developed breast recurrence, though age, initial stage, interval, treatment, and outcome for that specific patient were not separately reported.

Hsieh et al. [15] (2012): A single case of cervical SCNEC with breast metastasis detected on PET-CT, occurring alongside adrenal gland involvement in a disseminated presentation; age, initial stage, treatment, and outcome were not detailed in the available report.

Yadav et al. [16] (2015): A 42-year-old woman with FIGO stage IIIB cervical SCNEC (biopsy-confirmed, chromogranin- and synaptophysin-positive), treated with chemoradiation; bilateral breast metastases were discovered on follow-up PET-CT alongside disseminated bone and liver disease, and she was managed palliatively before discharge to hospice care.

Kwak [5] (2018): A 51-year-old postmenopausal woman with a 9-cm cervical mass and no distant metastases at diagnosis, treated with radical hysterectomy, bilateral salpingo-oophorectomy, and adjuvant pelvic radiotherapy; a palpable breast metastasis developed three months after surgery, treated with simple mastectomy and adjuvant chemotherapy. She initially responded but ultimately died of multi-organ metastatic disease with a fulminant course.

Wannasri et al. [11] (2025): Breast involvement one year after chemoradiation for the primary tumour, alongside disseminated lung, skin, peritoneal, and nodal disease, with a rapidly fatal course; age, initial stage, and breast-specific treatment were not detailed in the available report.

Chan et al. [17] (2003) and Shin and Caraway [18] (1998): Additional cases of breast metastasis in cervical small-cell carcinoma noted within a multivariate prognostic-factor series and a cytopathology review, respectively, without individual age, stage, treatment, or outcome detail reported for those cases.

Present case: A 29-year-old woman with FIGO stage IB2 cervical SCNEC (reclassified to IB3 on final surgical pathology), treated with radical hysterectomy and adjuvant chemoradiotherapy; an isolated breast metastasis developed three years later with no other systemic disease on PET-CT, treated with nipple-sparing mastectomy and adjuvant platinum-etoposide chemotherapy, achieving durable remission at 18 months.

Where reported, ages ranged from 42 to 51 years, and intervals to breast involvement ranged from three months to one year, with outcomes predominantly disseminated and fatal. By contrast, the present case had a considerably longer disease-free interval, an isolated presentation, and durable disease-free survival.

## Conclusions

Breast metastasis from cervical SCNEC is exceptionally rare and can closely mimic primary breast cancer on both clinical examination and imaging. Diagnosis relies on correlating the patient's oncologic history with morphology and immunohistochemistry, particularly the combination of neuroendocrine-marker positivity, absence of an in situ component, and negativity for breast-lineage markers. This case also illustrates that an isolated, oligometastatic presentation with a long disease-free interval does not necessarily carry the same grim prognosis as the rapid, multi-organ relapses described in earlier reports. In such selected patients, individualised management combining local surgical control with systemic chemotherapy, together with long-term surveillance, may offer a meaningful period of disease control.
